# Decline in body size and female fraction in the grass snake (Natrix natrix, Linnaeus 1758) population after 40 years (Southern Poland)

**DOI:** 10.1007/s11356-021-16128-y

**Published:** 2021-09-06

**Authors:** Stanisław Bury, Bartłomiej Zając, Henryk Okarma, Aleksandra Kolanek

**Affiliations:** 1 Department of Comparative Anatomy, Institute of Zoology and Biomedical Research, Jagiellonian University, Gronostajowa 9, 30-387 Kraków, Poland; 2 NATRIX Herpetological Association, Opolska 41/1, 52-010 Wrocław, Poland; 3 Institute of Environmental Sciences, Jagiellonian University, Gronostajowa 7, 30-387 Kraków, Poland; 4 Institute of Nature Conservation, Polish Academy of Sciences, Mickiewicza 33, 31-120 Kraków, Poland; 5 Department of Geoinformatics and Cartography, Institute of Geography and Regional Development, University of Wroclaw, pl. Uniwersytecki 1, 50-137 Wrocław, Poland

**Keywords:** Body size, Sex ratio, Reptile, Ectotherm, Decline, Global change

## Abstract

Depletion of free-living populations is often associated with changes in fitness-related traits, e.g., body size. Ongoing decrease in body size has been reported in most vertebrates, but reptiles remain understudied. Moreover, sexual size dimorphism, commonly observed in reptiles, indicates that environmental pressures on body size may appear sex-specific. This can also result in shifts in sex ratio, an aspect even less studied. We investigated body size and sex ratio in population of grass snake (*Natrix natrix*) surveyed over 40 years ago in comparison with the current state. We found that both sexes express similar magnitude in body size decline. The current sex ratio does not deviate from 1:1, while in the past, females outnumbered males. The observed changes are likely an outcome of several non-mutually exclusive factors. In the studied area, an increase in road traffic and human presence and a drop in prey availability have been documented. Both factors may exert higher pressure on larger individuals, particularly females, due to their high costs of reproduction. It is recorded here that increase in ambient temperatures and summer duration may additionally enhance the mortality risk and resource requirements. Shifts in body size and sex ratio can catalyze further declines in abundance and reproductive potential of the population.

## Introduction

The worldwide decline of wildlife is well documented and represents one of the major challenges to the conservation sciences (e.g., Butchart et al. 2010; Potts et al. 2010; Brashares et al. 2014). Loss of diversity and decline in population size are observed in all vertebrate taxa (e.g., Burbidge and McKenzie 1989; Gibbons et al. 2000; Schipper et al. 2008; Allentoft and O’Brien 2010). Therefore, it is crucial to recognize patterns associated with the decline process to predict its future dynamics. A drop in population numbers can be predicted to be preceded by alteration in population structure and individuals’ properties related to survival and reproduction (Stephens et al. 1999; Kurek et al. 2019). However, long-term data to investigate such changes are still relatively uncommon, especially for ectotherm vertebrates.

Body size is a trait fundamentally relevant in terms of fitness (Ziółko and Kozłowski 1983; Kozłowski 1992). It is well established that body size positively correlates with fecundity, e.g., larger females bear more offspring thanks to greater body cavity and fat stores (Olsson 1993; Shine 1994). Similarly, individual survival can be positively influenced by the body size which can be mediated by the amounts of energy reserves necessary to cope with dynamic ambient conditions (Civantos et al. 1999; Angilletta et al. 2004). Due to its association with fitness, the body size can potentially correlate with the whole population dynamics (Brose 2010; Russell et al. 2011). Alterations in body sizes over past decades have been well documented and are mainly visible as a decrease rather than an increase in size (Gardner et al. 2011). Such a decline in body size is reported repeatedly in endotherms, while ectotherm vertebrates remain less recognized (Gardner et al. 2011). Only recently has the body of data on ectotherms, especially fish and amphibians, increased indicating a similar decline in body size as in endotherm species (Bradshaw et al. 2008; Audzijonyte et al. 2013; Caruso et al. 2014), but studies showing the opposite trend have also been published (Tryjanowski et al. 2006). Data on reptiles is more underrepresented despite reptiles constituting a particularly interesting model due to very common sex-specific variation in body size (Shine 1994).

Sexual size dimorphism may drive sex-specific decline in body size, due to higher pressure on larger specimens (López-Calderón et al. 2017; Bury and Zając 2020). At the whole population level, size-biased pressure can result in changes of sex ratio. Temporal deviations in sex ratio have recently been recognized as likely to precede population decline (Kurek et al. 2019), but long-term changes in sex ratio represent an aspect even less recognized. It is therefore premature to conclude about the global patterns of the temporal changes in body size and sex ratio, but such data is critical to gain insight into the patterns associated with the global decline of reptile populations.

Here we studied the patterns of body size and sex ratio in a grass snake population in comparison to previous data from the same area obtained over 40 years ago (Juszczyk 1974). We predict that the average body size is currently lower compared to the previous time period as indicated by available studies (e.g., Gardner et al. 2011). Due to clear sexual size dimorphism in this species, with females attaining larger body size (Thorpe 1989; Madsen and Shine 1993), we expect that the magnitude of decline is higher in females. We also investigated the patterns in sex ratio, for which we expect a reduction in the female share over time, due to the anticipated higher pressure on larger specimens being enhanced in females by higher costs of reproduction. In addition we have explored possible environmental changes in the study area by interviewing Niepołomice Forestry Department (NFD) and quantifying indices of climate change at the local scale.

## Methods

### Study area and historical data

We have compared data on the body size and sex ratio in the population of European grass snake (*Natrix natrix*) inhabiting Lesser Poland (S Poland) from before 1974 (Juszczyk 1974) with its current state. Historical data in Juszczyk (1974) were collected in Puszcza Niepołomice Forest and in the vicinity of Cracow, but the exact timeframe of data collection is not provided; thus, we have arbitrarily assumed one decade before Juszczyk’s publication. New data were collected by us between 2015 and 2020, similarly in the Niepołomice Forest and in one site in the vicinity of Cracow with habitat that remained undestroyed over since the earlier time. In our previous study, we have already proven that snakes from these two sites do not express differences in their body size (Bury and Zając 2020); thus, we have pooled data collected in both sites into one dataset.

### Fieldwork

Snakes were captured year-round in both sites in parallel, measured, sexed based on the morphology of tail and cloacal region as well as individually marked with scale clipping (Brown and Parker 1976). Only sub-adult to adult snakes (> 35 cm of total length) were considered in our survey, as in Juszczyk (1974). Although Juszczyk included individuals with damaged tail, we decided to remove such specimens from the current survey. Such approach is more conservative, because we are not able to assess the frequency of individuals with damaged tail in Juszczyk’s dataset, and possible differences in their frequency between past and current dataset could lead to a bias in total size estimation.

### Data analysis

In the analyses we have used the data on total length, instead of snout-vent length, since only the total length was provided by Juszczyk. Data in Juszczyk (1974) was presented as the number of specimens in a given size class, without mean values and standard deviations. Therefore, for each size class, we produced a dataset that consisted of a given number of individuals, each with the size of a median value for a given size class. An exception to that were minimum and maximum values of total body length, because these particular values were provided by Juszczyk (1974). Such an approach reduces the variation and thus may seem to result in a high number of errors compared to the original dataset, but the sample size is large enough (*N* =398; Juszczyk 1974) to assume that estimations of mean values and dispersion are close to the original dataset. We have analyzed the length of snakes using two-way ANOVA that included study period (Juszczyk’s and our study) and sex (males and females), as well as interaction term. The sex ratio for both study periods was compared with the random one (1:1) using chi^2^ test. In total 398 individuals (153 males and 245 females) from Juszczyk’s study and 75 individuals (34 males and 41 females) from our study were used in the analysis of body length. For the analysis of sex ratio, we used the same sample size in the case of Juszczyk’s data, but larger (40 males and 45 females) in the case of our study, since we have added also specimens that exhibited loss of the tail tip.

Climatic data came from the E-OBS dataset (Cornes et al. 2018), downloaded as multidimensional NetCDF files (containing average daily temperatures and daily precipitation sums for 0.1 deg regular grid). Data was processed and exported using the R program to a spreadsheet for location 50.01 latitude, 20.27 longitude (Niepołomice Forest). Afterwards, for individual years from 1964 to 1973 and from 2010 to 2018, the length of the thermal summer (number of days with average temperature higher than 15°C) and average annual temperature were counted. We have chosen both periods arbitrary, since Juszczyk did not specified his study period. We assumed that a decade is likely to be a good representation of the conditions that could have affected the population parameters we obtained.

We have interviewed NFD in regard to the density of road web and road types in Niepołomice Forest, as well as the intensity of human presence associated with recreation activity in this area.

## Results

**Fig. 1:**
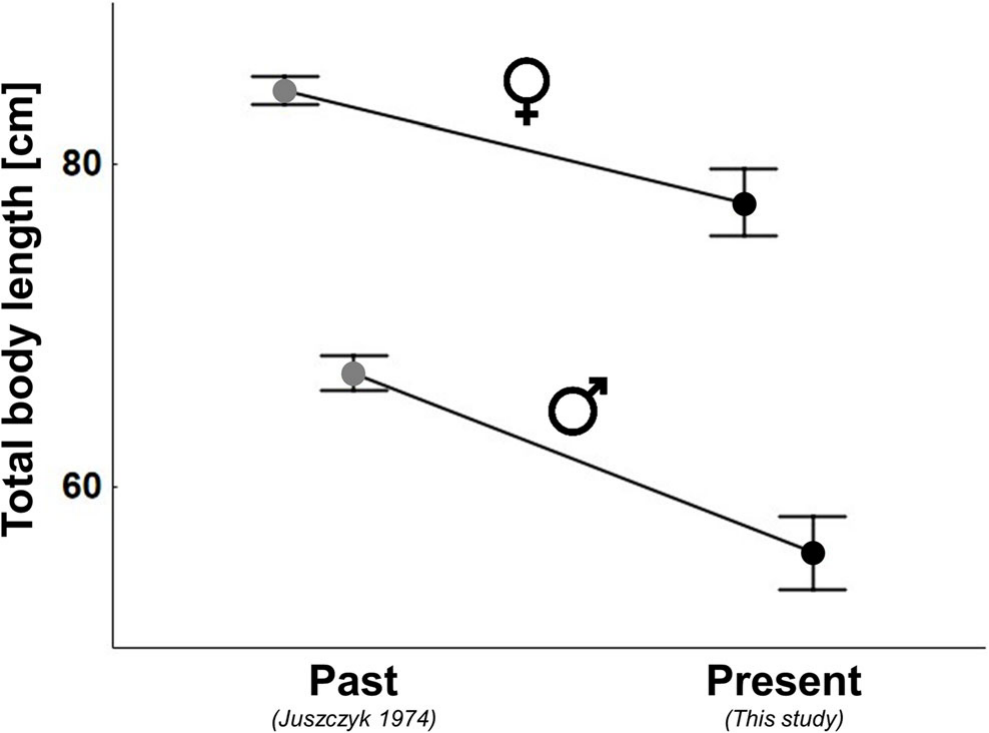
The effect of study period and sex on the total length of the grass snake (*Natrix natrix*) (data presented as LSM ± SE). Significant differences occur between both sexes and both study periods ”)

**Fig. 2:**
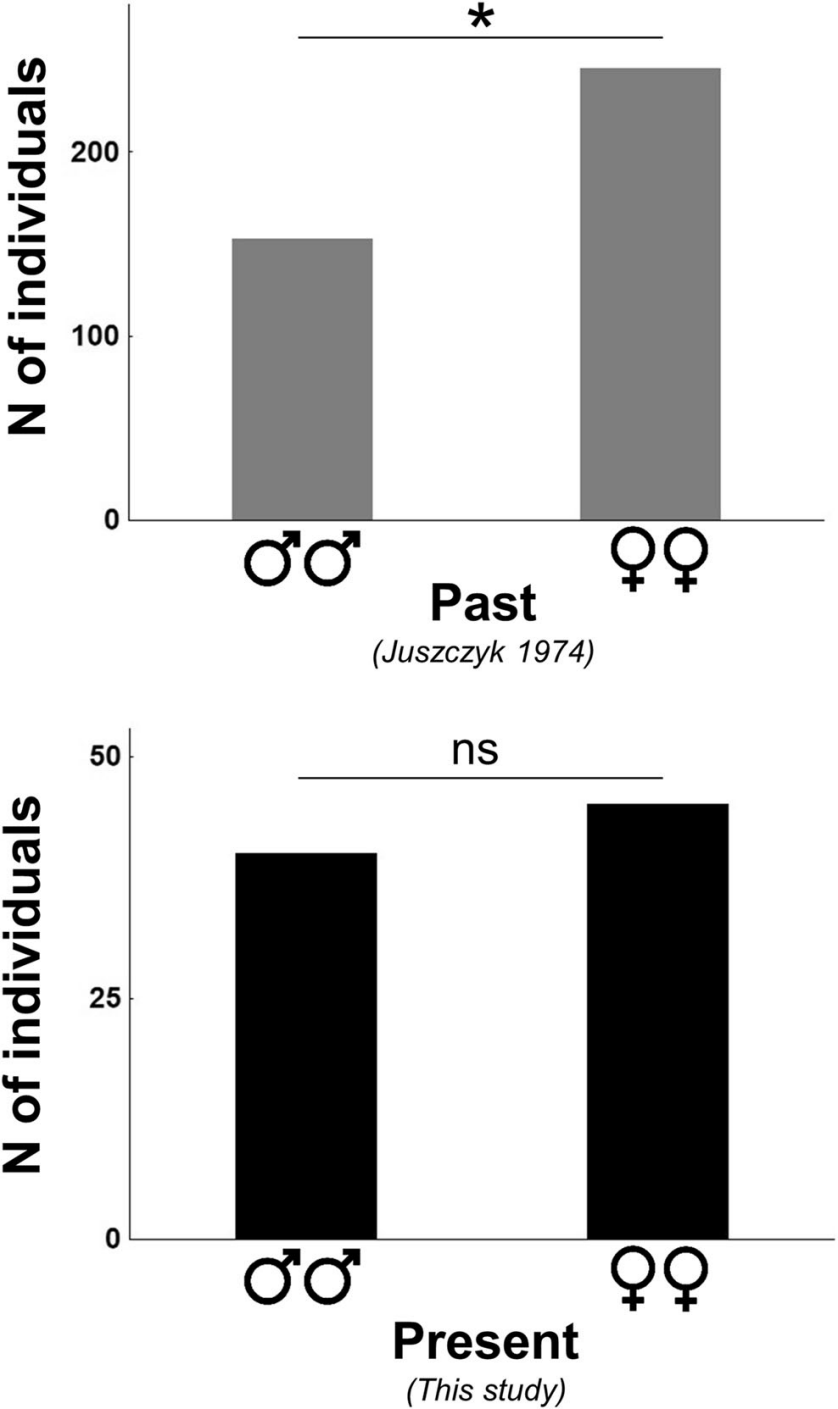
Sex ratio of the grass snakes population in both study periods. In the past females significantly outnumbered males, while currently the share of both sexes does not deviate from 1:1 ”

We have found a significant effect of the study period (*F*_1,469_=28.802; *p* < 0.001) on the body size in both males and females, i.e., decline in the total size over time  (Fig. 1). The effect of sex was also significant (*F*_1,469_=135.285; *p* < 0.001), but both factors, sex and time period, did not interact (*F*_1,469_=1.621; *p* =0.204). This indicates that size differences between the current and the past state are of comparable magnitude in both sexes. Although large specimens exceeding 100 cm and even reaching 120 cm are still observed, the average size of females has declined by 8.17% (from 84.56 ± 14.33 cm to 77.65 ± 17.53 cm), while in males by 16.68% (from 67.09 ± 10.65 cm to 55.90 ± 9.738 cm). In the past sex ratio was female biased (1:1.6; chi^2^=21.27; *p* < 0.001), while currently it does not deviate from 1:1 (1:1.125; chi^2^=0.294; *p* = 0.59), indicating that the share of both sexes has become equalized (Fig. 2).

Climatic conditions showed clear change over time. Specifically, between both study periods (1964–1973 vs 2010–2018), the average annual temperature rose (*t*=4.52; df=17; *p*=0.0003) by 1.63°C, while the average duration of climatic summer increased (*t*= −3.8; df=17; *p*=0.0014) by 25.48 days.

Reviews done with NFD did not provide quantitative results but rather descriptive information. As reported by NFD, the road network remains unchanged in the Niepołomice Forest since the 1960s–1970s. However, a large number of dirt roads have been covered with asphalt, and the traffic has considerably increased, but no quantitative data is available. While regular survey has been done to quantify the number of visitors to NFD, according to NFD officials, Niepołomice Forest has become a major recreational area in the neighborhood of Cracow since the beginning of the twenty-first century, and especially since 2010. Data on official recreational events (e.g., marathons) may hint at the magnitude of this change. For example, in 2013 three events occurred with ca. 800 participants in total, while in 2019 the number of such events increased to 16 with 5900 participants in total. Also, settlements around Niepołomice Forest are continuously spreading, including construction of whole new housing estates on former arable land and meadows close to forest. Therefore, the magnitude of increase in human presence is considered even higher over the period between the 1960s and currently.

## Discussion

Our study shows clear alterations in the grass snake population after a 40-year period. As expected, a decline in body size was observed, but contrary to our prediction, the magnitude of this decline appeared similar for both sexes. Three methodological considerations related to this finding need to be mentioned. Firstly, a clear discrepancy between Juszczyk’s and our datasets is visible in terms of sample size. Lower capture success in our survey may represent an outcome of possibly reduced abundance of snakes (Głowaciński and Sura 2018; Hagman et al. 2012) but is unlikely to affect direction of the obtained pattern. This is because short-term surveys, such as ours, tend rather to overestimate, not underestimate, the body size of a sample in case of snakes (Prior et al. 2001). Secondly, we refrained from using individuals that expressed the loss of tail tip, despite Juszczyk included such specimens. Therefore, the total size of snakes in Juszczyk’s sample is probably underestimated, which does not refer to our current survey. In other words, the actual difference between current and past body size can be even higher than here reported. Finally, an inter-observer variation in the accuracy of SVL assessment might have impacted our results. However, the magnitude of such errors is rather small, for example, in common lizards (*Zootoca vivipara*) they reach up to 3.5 % of SVL equaling to 2 mm (Roitberg et al. 2011). This can potentially be even smaller in larger species, such as grass snake, and is far below the documented here differences between Juszczyk’s and our study. Collectively, the declined body size can be considered a reliable and conservative result, because the abovementioned factors can either reduce or only negligibly influence the magnitude of the observed effect, with no impact on its trajectory. Next to the body size, we have observed alterations in sex ratio—as predicted the pattern in sex ratio points to a rather asymmetric response of males and females. While 40 years ago females outnumbered males, the sex ratio currently is equalized. This may result from an increased number of males or a reduced share of females. However, we perceive the latter as more likely in view of the well-documented decline in snake populations worldwide (Reading et al. 2010), which may be preceded by female loss (Kurek et al. 2019). Both the reduced body size and lower share of females may represent an outcome of similar factors and drive future dynamics of the population.

We have identified three factors likely to underlie smaller body sizes of both sexes and changed sex ratio. Firstly, the intensity of traffic has increased over the last 40 years, despite the network of roads in the study area remaining relatively unchanged (NFD pers. comm.). Such increased traffic could directly increase road mortality, which can sometimes quickly eradicate larger individuals (Andrews and Gibbons 2008). Also, the growing abundance of people in snakes’ habitat may cause an increase of human-snake encounters, which amplifies the mortality of larger individuals caused by intentional killing. Secondly, the population of amphibians (the main prey type of the grass snake, constituting 100% of its diet in Niepołomice Forest according to Juszczyk 1974) in the study area expressed a very strong decline in biomass and abundance compared to the 1960s (Pabijan et al. 2019). Food scarcity is known to impair growth and thus can constrain the eventual size achieved by an organism (Madsen and Shine 1993; Madsen and Shine 2000). Both factors seem to impact males and females similarly, as indicated by comparable body size decline in both sexes. However, the reduced share of females is currently compared to the past points to their higher vulnerability. This can be attributed to higher reproductive investment in females. Specifically, limited resource availability may decrease post-partum survivorship (Sperry and Weatherhead 2009), while high movement rate during oviposition additionally elevates road mortality (Bonnet et al. 1999). Our results corroborate previous findings on snakes (Bury and Zając 2020; Kurek et al. 2019) indicating a rather asymmetric response of both sexes towards environmental change.

The third factor we perceive as important refers to change in climatic conditions. Climate change, i.e., overall increase in ambient temperatures, is commonly said to underlie shifts in body size (Gardner et al. 2011; Sheridan and Bickford 2011) and is also clearly visible in our study area. Although temperature-size rule states that higher temperatures promote smaller body size (Walters and Hassall 2006), we do not perceive elevated average temperatures as likely to explain the pattern observed. Firstly, high environmental temperature does not translate into high body temperature, since the latter is maintained at certain optimal level through behavioral thermoregulation (e.g., Goller et al. 2014). Sufficient heterogeneity of the habitat to allow efficient thermoregulation may thus buffer the effect of temperature rise. Our study sites were located in wooded areas, so that a high level of thermal heterogeneity is secured (Pincebourde et al. 2007). Secondly, the empirical data on squamates suggests the opposite pattern to that predicted based on temperature-size rule, since in snakes and lizards, larger sizes are often observed in environments characterized by higher ambient temperature (Ashton and Feldman 2003). Instead of temperature rise per se, it is rather the associated extension in annual activity season due to prolonged climatic summer which is more likely to contribute to the observed changes. Specifically, a longer period of activity may enhance the impact of the abovementioned external mortality and food scarcity (Adolph and Porter 1996). An extended activity season means longer time of high energy turnover. This, in turn, imposes high requirements for resources, which are not necessarily met by the prey availability in the environment (Pabijan et al. 2019). Moreover, longer activity period equals extended exposure to mortality risk, e.g., associated with human presence and road traffic, both elevated over time (see above). Overall, we propose that the major impact of the climate warming on free-living reptiles acts indirectly, through its effect on external mortality and energy budgets.

Decrease in body size, similarly to alterations in sex ratio, may represent a trend preceding population decline due to its feedback effect on population dynamics (Kurek et al. 2019; Bury and Zając 2020). Firstly, smaller sizes reduce fecundity and survival (Civantos et al. 1999; Pincheira-Donoso and Tregenza 2011), thus population growth and persistence (O’Grady et al. 2004; Böhm et al. 2016). Secondly, smaller sizes reduce the capacity to cope with limited food and water resources (Winne et al. 2010). This is particularly important in view of ongoing extension of annual activity season along with more common periods of drought and food scarcity. We propose that size-dimorphic species may express higher susceptibility towards ongoing change, due to the differential pressure imposed on both sexes and associated deviations in sex structure. Further studies on a wide array of species are necessary to assess the susceptibility of different evolutionary lineages towards anthropogenic change.

## Data Availability

Data are available upon request.
